# Parents’ satisfaction on dental care of Dutch children with Autism Spectrum Disorder

**DOI:** 10.1007/s40368-020-00586-y

**Published:** 2021-06-18

**Authors:** L. S. Kind, I. H. A. Aartman, M. C. M. van Gemert-Schriks, C. C. Bonifacio

**Affiliations:** 1 Department of Cariology, Endodontics and Pedodontology, Academic Centre for Dentistry Amsterdam (ACTA), Gustav Mahlerlaan 3004, 1081 LA Amsterdam, The Netherlands; 2 Department of Social Dentistry and Behavioural Sciences, Academic Centre for Dentistry Amsterdam (ACTA), Amsterdam, The Netherlands; 3 Department of Pediatric Dentistry, Academic Centre for Dentistry Amsterdam (ACTA), Amsterdam, The Netherlands

**Keywords:** Dental care, Dentistry, Autism, Special needs

## Abstract

**Purpose:**

To assess if Dutch children with Autism Spectrum Disorder (ASD) regularly visit a dentist and to evaluate parent’s satisfaction on the care provided.

**Methods:**

Parents of ASD children (2–18 years) were invited to fill out a survey. The survey consisted of questions regarding ASD severity, frequency of dental visits, history of dental pain, type of dental practice and parents’ satisfaction. Results were analysed using Chi square and Mann–Whitney *U* tests (*α* = 5%).

**Results:**

Of the 246 returned questionnaires, 19 were excluded (incomplete or unconfirmed ASD diagnosis). All children visited a dentist at least once and 5% of them had their last visit more than 12 months ago. According to parents, 15% of the children did not receive the needed care when they had toothache and 21% of the parents were unsatisfied with the current dental care provided. No difference was found between satisfied and unsatisfied parents in type of dental practice visited (*p* > 0.05). The children of unsatisfied parents reported more often pain during the last year (*p* = 0.013) and had a more severe type of ASD (*p* = 0.016).

**Conclusions:**

The majority of Dutch ASD children investigated regularly visit a dentist and 21% of the parents is unsatisfied with the dental care provided.

## Introduction

There is an absence of research on the prevalence of Autism Spectrum Disorder (ASD) for the Dutch population. International research estimates the prevalence around 1% of the world population and the Dutch National Health Council assumes that the prevalence in the Netherlands to be the same. Following this assumption; there are approximately 160.000 people in the Netherlands with ASD (Williams et al. 2006). There is a substantial dispersion within the ASD people regarding skills, intelligence and treatability. However, there are also communalities: people with ASD often have difficulties with social interaction and communication, and exhibit typical patterns of behaviour and interests. Intellectual disability occurs in 60% of the ASD patients (Friedlander et al. 2003). An equal percentage has behavioural problems such as temper tantrums, hyperactivity, short attention span, anger and aggressiveness, or other co- morbid disorders such as epilepsy and gastrointestinal problems (Friedlander et al. 2003). People with ASD have difficulty coping with stress and often have anxiety disorders (Blomqvist et al. 2014). Also, unusual responses to sensory stimuli are very characteristic for the disorder, i.e., 70–90% of ASD patients is hypo- or hyper-sensitive to touch, smell, sound, taste or light (Stein et al. 2013).

ASD is not associated to specific intraoral characteristics, but patients do have features that are related to oral health. Many patients have difficulties swallowing food, which results in a longer presence of the food in the oral cavity (Önol and Kirzioglu 2018). In addition, there is often a preference for sweet or sticky foods. Cariogenic foods are often used as a reward for reinforcement of good behavior. All these factors increase the risk for dental caries in the ASD population (Loo et al. 2008). The rates of caries prevalence in ASD patients vary in the literature (Da Silva et al. 2017). Only a few studies are available about the caries prevalence in ASD children and they generally have small samples. Some authors mention that the prevalence of caries in ASD children is lower than in healthy developing peers (Loo et al. 2008; Fakroon et al. 2015), others report a similar or higher caries prevalence in children with ASD (El Khatib et al. 2014; Delli et al. 2013). Nevertheless, consensus does exist on the extent of treatment, stating that ASD children have untreated dental decay more often (Tchaconas and Adesman 2013; Kopycka-Kedzierawski and Auinger 2008). In addition, due to inadequate oral hygiene, children with ASD manifest gingivitis and periodontal disease more often than those without ASD (Pilebro and Backman 2005; Ferrazzano et al. 2020).

It can be assumed that due to the factors mentioned above, challenges in the dental setting are aggregate for both the dental care professional and the ASD patient. This assumption is supported by experience, whereas various studies also report upon the uncooperative behaviour, anxieties and negative experiences the children with ASD exhibit in the dental setting (Loo et al. 2008; Barry et al. 2014; Duker et al. 2017). Due to the mutual difficulties faced by both the dental caregivers and the ASD patients, dental health care is often lagging in children with ASD.

In the USA, 15% of the children with ASD reported to have unmet dental care needs versus only 6% of USA children overall (McKinney et al. 2014). Lai et al. reported in 2012 comparable results, 12% of the ASD children had unmet dental needs and 7% of the ASD children had never visited a dentist (Lai et al. 2012). Brickhouse et al. reported in 2009 that 19% of USA children with ASD had unmet dental needs and they showed that children with ASD who exhibit behavioural problems are less likely to receive regular dental care (Brickhouse et al. 2009). More recently, a study performed in Hong Kong also found significant differences in reported barriers to access to dental services among preschool children with and without ASD (Du et al. 2020). Their main complaints are the difficulty in finding a dentist who is willing to treat the ASD child or that the child cannot be cooperative. In this sense, children with ASD may be at risk of receiving inappropriate dental care, due to a lack of appropriately trained general dentists. It has been reported that, in the USA, as low as 10% of practitioners treated children with special needs on a regular basis (Casamassimo et al. 2004).

The studies mentioned above, all focus on the dental health care situation in the USA and China but are not generalizable to the Netherlands, where (dental) health care systems are differently organised. For example, costs of dental care or insurance have been cited as core barriers to dental care in USA and China. This does not count for the Dutch situation, due to the provision of free dental care for children up to 18 years. Furthermore, in the Netherlands, ASD children have the opportunity to go to different types of dental practices, i.e., the general dental practice, the pediatric referral dentist or the special dental care centre, often situated in a hospital setting. To be eligible for treatment in a pediatric referral clinic or special dental care centre, children need to be referred by, for example, a general dental practitioner, general practitioner or pediatrician. Although these factors could implicate that oral health care for ASD children is sufficiently covered in the Netherlands, no details are known and consequently no objective comparison to other countries can be made to evaluate if the current system is enough for the needs of ASD children. Therefore, the aim of this study is to examine if Dutch children with ASD regularly visit a dentist and to evaluate the satisfaction of their parents on the dental care provided.

## Materials and methods

### Ethical approval

The Medical Ethical Committee of the Vrije Universiteit Amsterdam declared that, according to the WMO (Dutch law for medical research involving human subjects), ethical approval was not needed.

### Participants

The sample comprised of parents/caregivers of children between 2 and 18 years with an ASD diagnosis who live in the Netherlands and who were willing to participate in this study. If parents had more children diagnosed with ASD, the survey had to be filled out considering the eldest child.

### Procedures

The invitation for filling out the survey was distributed through the websites of several ASD-communities, i.e., the Dutch Association for Autism (NvA/www.nva.nl), Autisme Paspoort (www.autismepaspoort.nl) and Autism friendly dentistry (www.autismevriendelijketandheelkunde.nl). All three communities referred to the survey in their digital newsletter, and the final two also placed the invitation on their Facebook page. The digital survey could be filled out via a link to Surveymonkey. As these were all free accessible websites with an unknown number of visitors that met the inclusion criteria, the response rate cannot be determined. This digital survey was accessible from November 2015 to February 2016. During the congress of the Dutch Association for Autism in November 2015, in the city of Utrecht, the Netherlands, parents and caregivers of children with ASD were invited face to face to fill out a paper version of the survey. The survey was filled out anonymously. There might be an overlap in parents visiting more than 1 available sites (i.e., website and/or congress). Therefore, in the consent letter parents were asked to fill out the survey maximum once.

### Informed consent

Before starting the survey, parents received information about this research. By filling out the survey they gave permission for using the responses in this research.

### Measures

After conducting a literature review on the barriers to dental care of patients with ASD and to examine the questions used in previous research about this subject, a new survey was designed, as there were no existing suitable surveys with questionnaires for this specific subject. The used survey was not validated as the main goal is to report parents’ satisfaction and to collect demographic data regarding ASD children dental visits and their parents’ satisfaction.

The survey consisted mostly of closed-ended questions, with space for further comment by the parents/caregivers*.* The survey included questions on basic demographics and questions regarding severity of ASD, frequency of dental visits, history of dental pain, type of dental practice (Tables 1 and 2) and parents’ satisfaction related to dental care provided.

**Table 1:** Basic features of the ASD children included (*N* = 227)

	*N*	%
*Child’s gender*		
Male	167	74
Female	60	26
*Child’s age (years)*		
2–6	20	9
7–12	116	51
13–18	91	40
*Childs’ perceived severity ASD*		
Severe	81	36
Moderate	109	48
Mild	37	16
*Other medical conditions*		
Yes	82	36
No	145	64

**Table 2:** Dental related features of the included ASD children (*N* = 227)

	*N*	%
*Child’s perceived dental health status*		
No teeth	1	0
Poor	6	3
Fair	72	32
Good	122	54
Excellent	26	11
*Child’s perceived cooperation in dental office*		
Really poor	45	20
Poor	35	15
Moderate	72	32
Good	51	22
Really good	24	11
*Challenges during last dental visit (multiple answers allowed)*		
Dentist didn’t treat my child well	46	20
My child wasn’t treatable	84	37
Other	11	5
No challenges	105	46
*Child toothache during the last 6 months*		
Yes	52	23
No	160	70
Don’t know	15	7
*Child received good dental care when in tooth pain (N* = *52)*		
Yes	42	81
No	8	5
Don’t know	2	4
*Type of dental practice*		
Regular practice	145	64
Pediatric dentist	54	24
Special needs dental center	25	11
Other	3	1

Statistical analyses were performed with SPSS, version 23. The collected data were compared using Chi-square and Mann–Whitney *U* tests. Significance was deemed at *p* < 0.05.

## Results

In total, 246 surveys were returned. Of these, 19 were excluded, because they were incomplete or the child did not have an ASD diagnosis confirmed. From the 227 complete surveys the characteristics of the children are included in Tables 1 and 2.

### Dental visits

All children (*N* = 227) had previous experience with a dentist. For 81% of the respondent ASD children, the last dental visit took place in the last 6 months. For 14% of them, the last dental visit was 6–12 months ago and for 5% of the children it was between 1 and 3 years ago. Only one child had his dental visit more than 3 years ago. Dental visit features for the parents of the respondent ASD children are: all of them reported to have experience with a dentist. Eighty-five percent of the parents had their last dental visit less than 6 months ago, 14% between 6 months and 1 year, 1% between 1 and 3 years and in 1% of the parents the last dental visit was more than 3 years ago.

### Oral hygiene

Regarding the oral hygiene, 49% of the parents found it difficult to brush their child’s teeth. Brushing frequency was for 4% of the children only 0–3 times per week, 22% brushed once a day, 23% brushed 8–11 times per week, and half of the children had their teeth brushed twice a day. Additionally, the children with severe type of ASD (*N* = 81) were found to be equally distributed between referral dental practices and general dental practice (*p* > 0.05). These children are also found to be significantly more often difficult to brush (*X*^2^ = 6.093, *df* = 1, *p* = 0.014). Furthermore, for children with severe type of ASD, the perceived cooperation in the dental chair is significantly worse, compared to that of children with the moderate or mild types of ASD (*X*^2^ = 11.176, *df* = 4, *p* = 0.025).

### Satisfaction on dental care

From the answers given to the posed questions, 21% of the parents is not satisfied with the dental care their child receives. The parents who were satisfied with the dental care provided to their child, were less likely to have problems with brushing their child’s teeth, compared to the dissatisfied parents (*X*^2^ = 5.993, *df* = 1, *p* = 0.014). Also, satisfied parents rated the cooperation of their child in the dental office higher (*p* = 0.001) and the perceived dental health status of their child was better than the rate of dissatisfied parents (*p* < 0.001), indicated by the Mann–Whitney *U* test. Additionally, the parents who were not satisfied with the dental care their child currently receive, were parents whose child more often had a toothache during the last year (*X*^2^ = 8.650, *df* = 2, *p* = 0.013), had more often a severe type of ASD (*X*^2^ = 5.809, *df* = 1, *p* = 0.016) and did not visit a dentist for more than 1 year (*p* < 0.001). The satisfaction of the parents is not related to the type of dental practice their child visited (*p* > 0.05), nor to the gender of the child (*X*^2^ = 0.727, *df* = 1, *p* > 0.05) and is not related to the child having a complementary diagnosis or not (*X*^2^ = 1.909, *df* = 1, *p* > 0.05).

### Last dental visit more than 1 year ago

When the child with ASD visited a special dental care center for his/her dental care, the chance for not visiting a dentist more than 1 year was significantly lower (*X*^2^ = 24.82, *df* = 3, *p* < 0.001), in comparison to the patients from a pediatric referral clinic or a general dental practice. Not visiting a dentist more than 1 year was not related to the gender of the child (*X*^2^ = 1.026, *df* = 1, *p* > 0.05), nor to the severity of ASD (*X*^2^ = 0.719, *df* = 1, *p* > 0.05). Remarkably, all children that had their last dental visit more than 1 year ago (*n* = 13), had a parent whose last dental visit was less than 1 year ago.

### Emergency dental care

The parents who answered that they could not find appropriate dental care when their child had a toothache, were parents of children who did not visit a dentist for more than 1 year (*X*^2^ = 12.012, *df* = 4, *p* = 0.015). Furthermore, not receiving the needed dental care was not related to gender (*X*^2^ = 1.107, *df* = 4, *p* > 0.05) nor to the severity of ASD (*X*^2^ = 1.213, *df* = 3, *p* > 0.05). These children were significantly more often patients in a general dental practice (*X*^2^ = 15.911, *df* = 6, *p* = 0.008) compared to the special dental care center and pediatric referral clinic.

## Discussion

Dental care remains the most prevalent unmet health care for children with special needs (Lewis et al. 2005). In addition, oral health-related quality of life was poorer among children with ASD, compared to children without ASD (Du et al. 2020). This study investigated if Dutch children with ASD regularly visit a dentist and if their parents were satisfied with the dental care provided. All investigated children in this study have visited a dentist at least once. Nevertheless, 21% of the parents report dissatisfaction with the dental care currently provided and for 5% of the children the last dental visit was more than 1 year ago, meaning that these children do not visit the dental office frequently. The finding that all children have dental experience could be explained by the population of parents involved in the present study. They can be considered motivated parents, because they are involved in ASD specific programs or participating in conferences or websites. This influences the external validity of the present study.

The general advise in the Netherlands is to visit the dentist twice a year for a dental check-up, and in case of ASD children this frequency can be higher with visits scheduled every 3–6 months. From the results of the present study, the frequency of dental visits of the ASD children in the last 12 months was higher in the Netherlands (95%) when compared to USA (71–88%) and China (75%) (McKinney et al. 2014; Brickhouse et al. 2009; Du et al. 2020). The differences in reimbursement by the insurance system could account for that. While in the USA, only few medical insurance plans include coverage for dental expenses, in the Netherlands, dental costs are mostly covered for all children up to 18 years old. Therefore, prosperity cannot be a reason for not visiting a dentist in the Netherlands.

As expected, the biggest problems were encountered for the children with a severe type of ASD. The results of this study indicated that children with a severe type of ASD were more difficult to brush and also more difficult to treat, in case dental treatment is indicated. According to the parents, the most cited problem during the child’s last dental visit is the child’s behaviour in the dental office. This finding is in accordance with previous studies (Du et al. 2020; Thomas et al. 2018).

General dentists may not be adequately equipped to handle the behaviour of this specific child population. In a multi-center survey in the USA, it was found that only 10% of the American general dentists often perform dental treatment to children with special health care needs (Lai et al. 2012). In the present study, the children with a higher rated level of ASD severity were equally treated by the general dentist relative to being treated by dentists in a special dental care centre or paediatric dental clinic. However, children whose cooperation is rated as low, are more often treated in these referral practices. Although it is expected that dentists in a special dental care centre or paediatric dental clinic are better able than the general dentist to handle the behaviour of children with ASD, these specialists have to deal with more difficult cases than the general dentist. This calls for further study upon this subject.

Further research is also required to investigate the dissatisfaction with the dental care received of the 21% of the parents. It should be deepened out what aspect of the dentist or the dental treatment they are specifically dissatisfied with. Due to the scope and type of questions contained in the survey used in the current study, the causes for dissatisfaction could not be specified.

From the results of this study, it appeared that children who had their last dental visit longer than 1 year ago, had a parent whose last dental visit was less than 1 year ago. It seems that having a child with ASD can be a barrier for frequent dental visits, even when the parent’s do visit the dentist themselves. Former research reported that children of mothers who did not visit a dentist regularly were at greater risk of not receiving dental care (Goettems et al. 2012), but in our study even the children of parents who do visit the dentist regularly could be considered at a relative risk of not receiving dental care. Isong et al. reported in 2010 that children were more likely to regularly visit a dentist when their parents also did (Isong et al. 2010). A possible reason for not visiting the dentist on a regular base could be related to the distance and accessibility of the practice. The distance can also be a plausible explanation for most of the children visiting general dental practice (64%) instead of pediatric dental clinics or special dental care centres (35%) as these centers are frequently not in a short distance from most of the family houses. Another explanation could be that parents avoid bringing their ASD child to the dentist, because they anticipate on a compromised cooperation of their child (Du et al. 2020).

With regard to the limitations of the present study, parents rated the severity of the ASD and the cooperation of the child during dental visits themselves. Parental reports could be biased and caregiver’s assessment of behaviour may or may not represent the actual behaviour of the child. Furthermore, there was no mention on parental education and special needs education, which can affect parental perception. On the other hand, Marshall et al. 2008 found that parents, in more than 88% of the cases, accurately predicted whether their ASD child would permit an examination in the dental chair (Marshall et al. 2008). The present cohort can be also considered as biased, because it is small and parents without access to internet or the congress were not reached.

## Conclusion

In conclusion, the results of this study indicate that, despite that all children in this research have visited a dentist at least once, dental care of Dutch ASD children is not optimal. Due to the vulnerability of this patient group and their specific needs, it is of utmost importance to perform further research upon dentists’ knowledge and skills, treatment options and effective behavioural management techniques.

## Data Availability

If needed, the corresponding author can provide details of data.

## References

[CR1] Barry S, O’Sullivan EA, Toumba KJ (2014). Barriers to dental care for children with Autism Spectrum Disorder. Eur Arch Paediatr Dent.

[CR2] Blomqvist M, Dahllöf G, Bejerot S (2014). Experiences of dental care and dental anxiety in adults with Autism Spectrum Disorder. Autism Res Treat.

[CR3] Brickhouse TH, Farrington FH, Best AM, Ellsworth CW (2009). Barriers to dental care for children in Virginia with autism spectrum disorders. J Dent Child.

[CR4] Casamassimo PS, Seale NS, Ruehs K (2004). General dentists’ perceptions of educational and treatment issues affecting access to care for children with special health care needs. J Dent Educ.

[CR5] Da Silva SN, Gimenez T, Souza RC, Mello-Moura ACV, Raggio DP, Morimoto S, Lara JS, Soares GC, Tedesco TK (2017). Oral health status of children and young adults with Autism Spectrum Disorders: systematic review and meta-analysis. Int J Paediatric Dent.

[CR6] Delli K, Reichart PA, Bornstein MM, Livas C (2013). Management of children with Autism Spectrum Disorder in the dental setting: concerns, behavioural approaches and recommendations. Med Oral Patol Oral Cir Bucal.

[CR7] Du RY, Yiu CKY, King NM (2019). Oral health behaviors of preschool children with Autism Spectrum Disorders and their barriers to dental care. J Autism Dev Disord.

[CR8] Du RY, Yiu CKY, King NM (2020). Health- and oral health-related quality of life among preschool children with Autism Spectrum Disorders. Eur Arch Paediatr Dent.

[CR9] Duker LIS, Henwood BF, Bluthenthal RN, Juhlin E, Polido JC, Cermak SA (2017). Parents' perceptions of dental care challenges in male children with Autism Spectrum Disorder: an initial qualitative exploration. Res Autism Spectr Disord.

[CR10] El Khatib A, Tekeya M, Tantawi MA, Omar T (2014). Oral health status and behaviours of children with Autism Spectrum Disorder: a case control study. Int J Paediatr Dent.

[CR11] Fakroon S, Arheiam A, Omar S (2015). Dental caries experience and periodontal treatment needs of children with Autistic Spectrum Disorder. Eur Arch Paediatr Dent.

[CR12] Ferrazzano GF, Salerno C, Bravaccio C, Ingenito A, Sangianantoni G, Cantile T (2020). Autism Spectrum Disorders and oral health status: review of the literature. Eur J Paediatr Dent.

[CR13] Friedlander AH, Yagiela JA, Paterno VI, Mahler ME (2003). The pathophysiology, medical management, and dental implications of autism. J Calif Dent Assoc.

[CR14] Goettems ML, Ardenghi TM, Demarco FF, Romano AR, Torriani DD (2012). Children’s use of dental services: influence of maternal dental anxiety, attendance pattern, and perception of children’s quality of life. Community Dent Oral Epidemiol.

[CR15] Isong IA, Zuckerman KE, Rao SR, Kuhlthau KA, Winickoff JP, Perrin JM (2010). Association between parents’ and children’s use of oral health services. Pediatrics.

[CR16] Kopycka-Kedzierawski DT, Auinger P (2008). Dental needs and status of autistic children: results from the National Survey of Children’s Health. Pediatr Dent.

[CR17] Lai B, Milano M, Roberts MW, Hooper SR (2012). Unmet dental needs and barriers to dental care among children with Autism Spectrum Disorders. J Autism Dev Disord.

[CR18] Lewis C, Robertson AS, Phelps S (2005). Unmet dental care needs among children with special health care needs: implications for the medical home. Pediatrics.

[CR19] Loo CY, Graham RM, Hughes CV (2008). The caries experience and behavior of dental patients with Autism Spectrum Disorder. J Am Dent Assoc.

[CR20] Marshall J, Sheller B, Mancl L, Williams BJ (2008). Parental attitudes regarding behavior guidance of dental patients with autism. Pediatr Dent.

[CR21] McKinney CM, Nelson T, Scott JM, Heaton LJ, Vaughn MG, Lewis CW (2014). Predictors of unmet dental need in children with Autism Spectrum Disorder: results from a national sample. Acad Pediatr.

[CR22] Önol S, Kirzioglu Z (2018). Evaluation of oral health status and influential factors in children with autism. Niger J Clin Pract.

[CR23] Pilebro C, Backman B (2005). Teaching oral hygiene to children with autism. Int J Paediatr Dent.

[CR24] Stein LI, Polido JC, Cermak SA (2013). Oral care and sensory over-responsivity in children with Autism Spectrum Disorders. Pediatric Dent.

[CR25] Tchaconas A, Adesman A (2013). Autism Spectrum Disorders: a pediatric overview and update. Curr Opin Pediatr.

[CR26] Thomas N, Blake S, Morris C, Moles DR (2018). Autism and primary care dentistry: parents’ experiences of taking children with autism or working diagnosis of autism for dental examinations. Int J Paediatr Dent.

[CR27] Williams JG, Higgins JPT, Brayne CEG (2006). Systematic review of prevalence studies of Autism Spectrum Disorders. Arch Dis Child.

